# Algae-mediated route to biogenic cuprous oxide nanoparticles and spindle-like CaCO_3_: a comparative study, facile synthesis, and biological properties

**DOI:** 10.1039/d1ra00187f

**Published:** 2021-03-11

**Authors:** Parisa Taherzadeh Soureshjani, Ahmad Shadi, Fatemeh Mohammadsaleh

**Affiliations:** Department of Biological Science and Technology, Faculty of Nano and Bio Science and Technology, Persian Gulf University Bushehr 7516913817 Iran shadi@pgu.ac.ir f.mohammadsaleh@gmail.com +98-077-31223350; Department of Chemistry, Faculty of Nano and Bio Science and Technology, Persian Gulf University Bushehr Iran

## Abstract

Biocompatible syntheses of Cu_2_O nanoparticles are relatively low compared to some other reported metal oxides due to their low stability and requiring more carefully controlled synthetic conditions. In the present study, the efficiency of three brown algae (*Cystoseira myrica*, *Sargassum latifolium* and *Padina australis*) extracts collected from the Persian Gulf was evaluated in the biosynthesis of Cu_2_O nanoparticles. A fast and simplified synthesis of Cu_2_O nanoparticles with average size between 12 and 26 nm was successfully achieved through an eco-friendly method using the aqueous extracts of *Sargassum latifolium* and *Cystoseira myrica*. Whereas, under the same reaction conditions using *Padina australis* extract no Cu_2_O nanoparticles were produced, and unexpectedly, the results approved the formation of spindle shaped CaCO_3_ with average sizes of 1–2 μm in length and 300–500 nm in width. Structure, morphology and composition of the as-prepared products were characterized by XRD, FT-IR, UV-vis, TEM and FESEM analysis. This work confirms that the biomolecules present in algae have the ability to affect particle size, morphology, composition, and physicochemical properties of the synthesized particles. The Cu_2_O nanoparticles prepared in this study were stable and exhibited efficient antibacterial and anticancer activity. This biosynthesis technique can be valuable in environmental, biotechnological, pharmaceutical and medical applications.

## Abstract 2

Cu_2_O nanoparticles were produced using aqueous extract of the brown algae featuring *Cystoseira myrica* and *Sargassum latifolium* and examined as anticancer and antibacterial agents.

## Graphical abstract

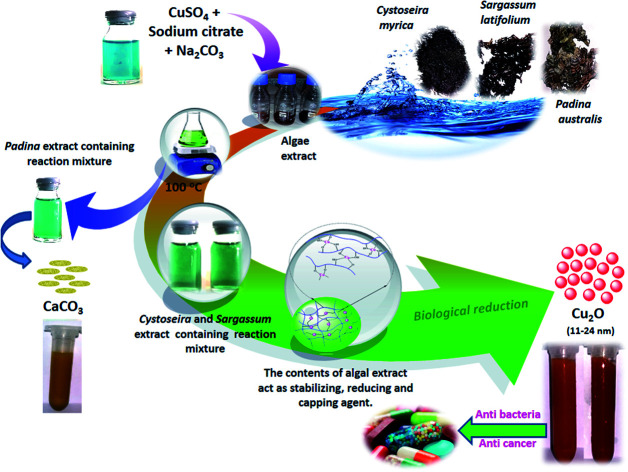

## 1. Introduction

Green nanotechnology has nowadays emerged as an area of research involving more eco-friendly and energy-efficient methodologies for the synthesis of metal-based nanoparticles.^1,2^ Biological synthesis of NPs has been proposed as an alternative to physicochemical synthesis because of the fundamental principles of ‘green’ chemistry and striving to have a clean world. It focuses on the fabrication of NPs using eco-friendly, harmless and commercially viable substances. The biological systems such as bacteria, viruses, algae, yeast, fungi and plants have been extensively used in these environment-friendly approaches.^3^ Simple methods have been established including of extracellular or intracellular reduction of metal ions by biological extracts.^4,5^ These extracts transform metal precursors to their corresponding NPs. Interestingly; noteworthy studies related to the biosynthesis of metal-based nanoparticles have focused on the use of various types of algae in the recent decade.^2,5,6^ The algal species have been widely considered as a powerful tool for green synthesis of inorganic nanoparticles with high efficiency, probably due to their high metal uptake potential. In addition, the convenient culture of algae, non-toxicity, low cost and easy availability are advantages which can make the study of algae-mediated biosynthesis of nanoparticles valuable and interesting. Current interest in these researches focuses on control of size, shape and composition of nanoparticles to manipulate their physicochemical properties.^7,8^

Cuprous oxide (Cu_2_O) is a p-type semiconductor which has potential applications in catalysts and photocatalyst,^9,10^ solar cells,^11^ pigments,^12^ and also has been used as a fungicide an antibacterial agent.^13,14^ Cu_2_O crystals with different morphologies have been prepared by chemical reduction approaches in which Cu^2+^ ions convert to Cu^+^ with a reducing agent and several successful methods have been reported for the production of Cu_2_O.^15–18^

Some papers have reported the green and convenient biosynthesis of different metal oxide nanoparticles such as Fe_3_O_4_,^19^ CuO,^20^ TiO_2_,^21^ ZnO,^22^ Cu_2_O/CuO^23^ and Ag/Cu_2_O,^24^ which were produced using natural products. However, up to now, there are relatively few reports on the bio-preparation and algae-mediated selective biosynthesis of nanocrystalline Cu_2_O compared with some other metal-based nanoparticles.^25,26^ The selective synthesis of stable Cu_2_O NPs remains an interesting task mainly because it is known that Cu_2_O is chemically unstable with respect to rapid air oxidation to form CuO. Therefore, a number of synthetic approaches have proposed the formation of a mixed Cu_2_O/CuO^27^ or Cu_2_O/Cu^13^ phase and it has widely recognized that the composition, size, and shape of the copper-based NPs can be altered by some controlling parameters of the reaction conditions such as time, temperature, pH, and the concentration of reagents as well as of stabilizing agents.

In recent years, various applications for nanostructured materials have been reported.^28,29^ The nanomaterials have shown significant potential for use in bactericidal applications.^28,30–32^ Multidrug resistance (MDR) is growing in both Gram positive and Gram negative bacteria and is compromising the effectiveness of antibiotic therapy especially by increasing the healthcare associated infections (HAIs) causing several thousands of deaths annually.^33,34^ The hope for development of new antibiotics has been diminished by the rapid resistance of microbial pathogens, reduced incentive to innovate new drugs and challenges related to drug development process. Nanostructured materials such as Zr/MoS_2_,^30^ Cu/TiO_2_,^31^ Zr/TiO_2_ (ref. 28) and Cu/ZnO^35^ have recently gained interest for their antibacterial and cytotoxic potential. Over the last years, copper oxide (CuO, Cu_2_O) nanoparticles (NPs) have gained considerable attention in biological applications. Several studies have described antibacterial activity of copper oxide NPs against Gram-positive and Gram-negative bacteria.^36^ Particularly, Cu_2_O NPs have been reported to show good environmental effects, lower toxicity, and significant antibacterial activity against the various bacteria through the production of reactive oxygen species (ROS) and release of copper ions.^37^ However, the biological properties of Cu_2_O nanoparticles having exclusive chemical and structural capabilities compared to CuO nanomaterials are comparatively less studied.

**Fig. 1:**
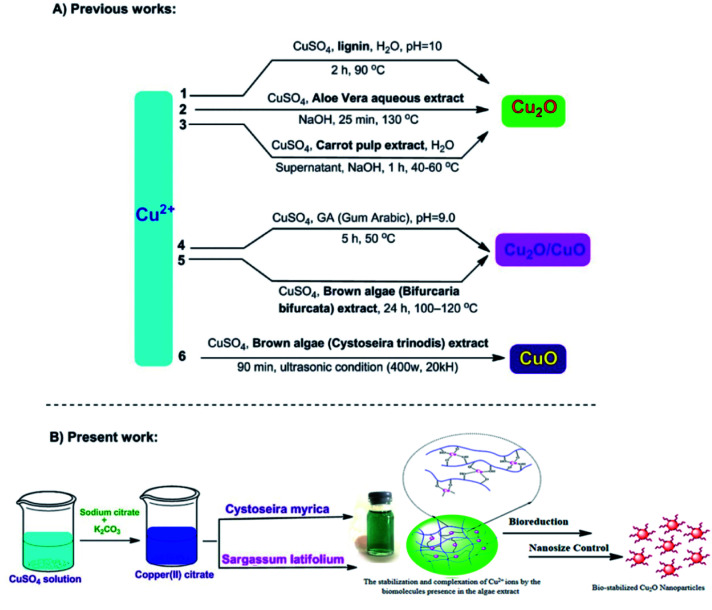
Different biosynthetic methods for the copper-based nanoparticles production; 1,^38^ 2,^25^ 3,^26^ 4,^23^ 5,^27^ 6.^39^

In the present work, we have examined the potential of three brown algae featuring *Cystoseira myrica* (*C. myrica*), *Sargassum latifolium* (*S. latifolium*) and *Padina australis* (*P. australis*) as biofactory for the synthesis of Cu_2_O nanoparticles (Fig. 1). Herein, we utilized the aqueous extract solution of these brown algae as reducing and stabilizing agents and to the best of our knowledge; this is the first comparative study of these algae in the selective biosynthesis of Cu_2_O nanoparticles. Detailed investigations on the influences of reaction time, temperature and reagent concentrations have been found in a solvothermal and controlled approach to synthesize the optimal Cu_2_O NPs, which are stable for several months. Subsequently, the as-prepared NPs were examined as anticancer and antibacterial agents.

## 2. Results and discussion

**Fig. 2:**
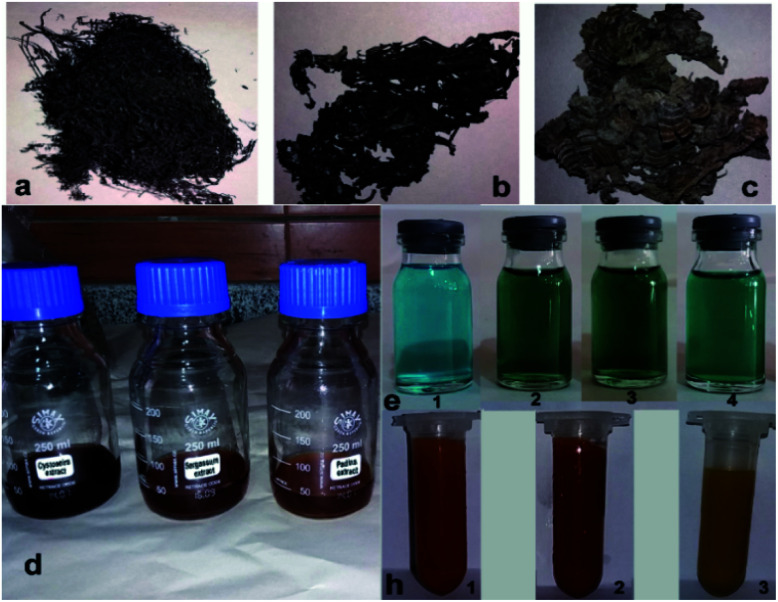
Dried microalgae: (a) (*C. myrica*), (b) (*S. latifolium*), (c) (*P. australis*). The algal crude extracts (d). CuSO_4_ solution (e-1) and the color change of reaction media after adding aqueous extracts of *C. myrica* (e-2), *S. latifolium* (e-3) and *P. australis* (e-4). Color of the particles produced using *C. myrica* (h-1), *S. latifolium* (h-2) and *P. australis* (h-3) extracts.

This study aimed to investigate the biosynthesis of Cu_2_O nanoparticles using the aqueous extract of three marine brown algae as reducing and capping agent. The algal crude extracts were a brown to dark-brown liquid (Fig. 2). In the beginning, after mixing the algal extracts separately with the blue solution of CuSO_4_·5H_2_O, no Cu_2_O nanoparticle was seen overnight at room temperature. Also, no nanoparticle formation was observed by repeating the same reaction under heating at 100 °C. The pH of reaction medium is one of the important experimental parameters in biogenic nanoparticle synthesis. Specially, a salient feature of biogenic synthesis is the ability of bio-active materials to operate under a special condition with the production of pure nanoparticles. According to literatures the synthesis of Cu_2_O nanoparticles is greatly affected by pH.^18^ These reports reveal that the shape, size, composition variation and overall reaction mechanism were commonly determined by the pH-dependent precursor species in the reaction media.

The pH of algal extract solutions studied in this work was found to be acidic, ranges about 4–5. When adding different concentrations of NaOH with different pH, the reaction solution changes from acidic to alkaline, however the nanoparticle formation was not seen in the reaction media at the same previous conditions (1 h, 100 °C).

The reaction conditions for the synthesis of Cu_2_O nanoparticles must be completely controlled because the Cu(0), CuO phase and other impurities may be formed in the reaction medium. The pH is an important parameter in our synthetic method, but it is not enough. We examined different pH values (4.0, 7.0, 9.0, 12) to find the optimal reaction conditions and the best results were obtained in the pH = 12 using K_2_CO_3_ base. However, no Cu_2_O nanoparticles were observed in the presence of NaOH in the reaction. By using NaOH, a dark brown solid was produced in the reaction mixture that the analysis of this solid did not show any signal of Cu_2_O product. Therefore, we found that the K_2_CO_3_ is an effective base in this reaction system. Without K_2_CO_3_, the blue color of the reaction mixture turned black, when we started heating.

After many experiments and observations, we developed a two-step reductive process to form nanoparticles and slow down the rate of nucleation and growth. For this, a deep-blue solution of deionized water, CuSO_4_·5H_2_O, sodium citrate and sodium carbonate was prepared. Then, algal extracts were separately added drop-wise into the deep-blue solution with continuous stirring at 100 °C. The pH of reaction mixture was measured to be about 12. After a short reaction time, the color change of reaction mixture and the crystallization of particles were clearly observed for all three reaction systems containing different algae extracts. The sodium citrate as a ligand reacts with Cu^2+^ to stabilize the Cu^2+^ precursor and facilitate its reactivity with algal capping and reducing agents.

After adding *C. myrica* and *S. latifolium* extracts, the reaction mixture changed from deep blue to a greenish appearance, and then, the orange and reddish Cu_2_O nanoparticles gradually were obtained, respectively, within half an hour with heating at 100 °C and stirring. At same reaction condition, the color of reaction mixture containing *P. australis* extract was changed to blue-green and then yellowish CaCO_3_ particles were produced in the reaction media (Fig. 2). The change in the color of the reaction mixture provides a convenient signature to indicate the production of particles in the reaction media. This product formation was initially confirmed visually and then by using XRD technique which have been frequently used to characterize the chemical phase of the metal-based nanoparticles.

### 2.1. Characterization of NPs

#### 2.1.1 XRD

**Fig. 3:**
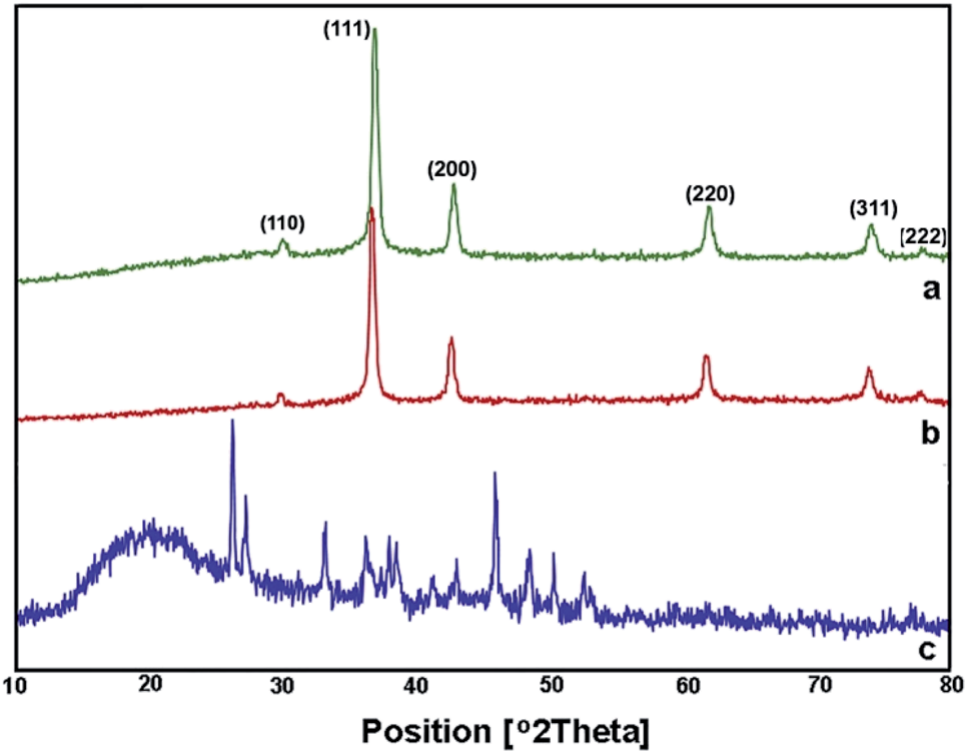
XRD spectra of Cu_2_O NPs produced using *C. myrica* (a) and *S. latifolium* (b) extracts. XRD pattern of particles produced in the presence of *P. australis* extract (c).

X-Ray reflective diffraction (XRD) analysis was carried out to get detailed information about the architecture and chemical phase of samples synthesized using three algae extracts. As demonstrated in Fig. 3, the XRD patterns of NPs obtained using both algae *C. myrica* and *S. latifolium* indicate diffractions of Cu_2_O particles, containing peaks that are clearly distinguishable and all of them can be perfectly indexed to Cu_2_O NPs. The peak positions are in good agreement with those for Cu_2_O powder reported in references (JCPDS 5-667).^18^ No characteristic peaks arising from Cu or CuO could be observed in the XRD patterns, indicating that pure Cu_2_O could be successfully obtained using these two kinds of algae extracts. However, the presence of different crystalline phases can be clearly seen in the XRD pattern of sample as-synthesized using the *P. australis* extract (Fig. 3), in which there is no phase of CuO and/or Cu_2_O. According to the reported ref. 40–42, all of the peaks were indexed to calcium carbonate (CaCO_3_) as either aragonite or calcite phase. It is well known that *P. australis* is one of the brown calcifying algae which precipitates CaCO_3_ in the microscopy form of Aragonite needle-like particles.^40,43^ The mineral compositions of the liquid extract of *P. australis* containing calcium (Ca^2+^) could form the CaCO_3_ phases through a mineral precipitation process.^41^ Our result is in well agreement with the XRD data reported by other groups.^40,41^

The first objective in the synthesis of metal-based nanoparticles is the choice of relevant capping agent and reducer. Indeed, the composition, size distribution and stability may be affected by the nature of reducing agent. The competent reducing agent produces the smallest, pure and stable nanoparticles in the reaction system.

#### 2.1.2 FE-SEM and TEM

**Fig. 4:**
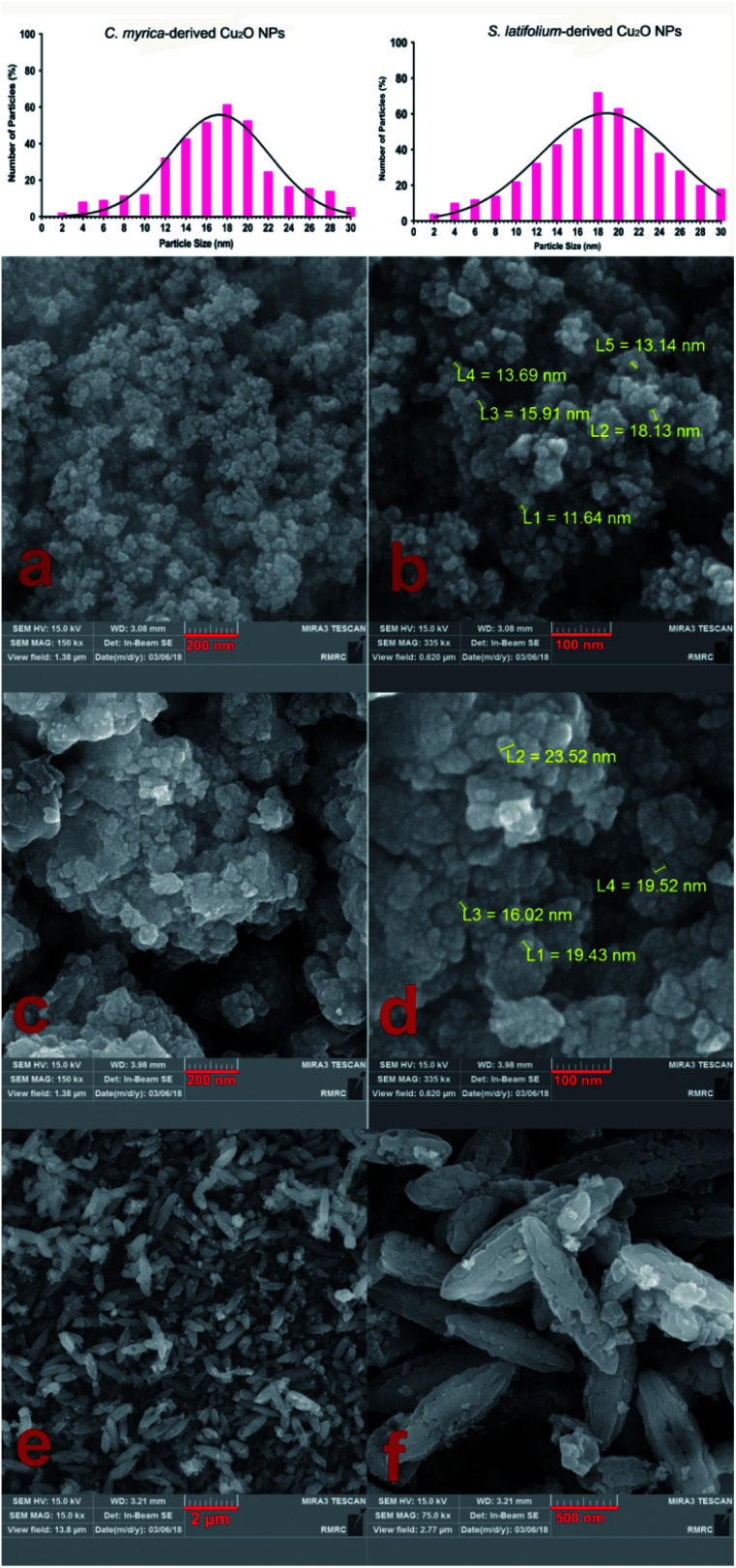
Particle size distribution diagram of the Cu_2_O NPs. FE-SEM images of the Cu_2_O NPs produced using *C. myrica* (a and b) and *S. latifolium* (c and d) extract. FE-SEM images of particles produced in the presence of *P. australis* extract (e and f).

The phase morphology and particle size measurement of the prepared samples were studied using field emission scanning electron microscopy (FE-SEM). As shown in Fig. 4(a–d), the FE-SEM micrographs of the samples produced using *C. myrica* and *S. latifolium* clearly depict nano-sized particles.

**Fig. 5:**
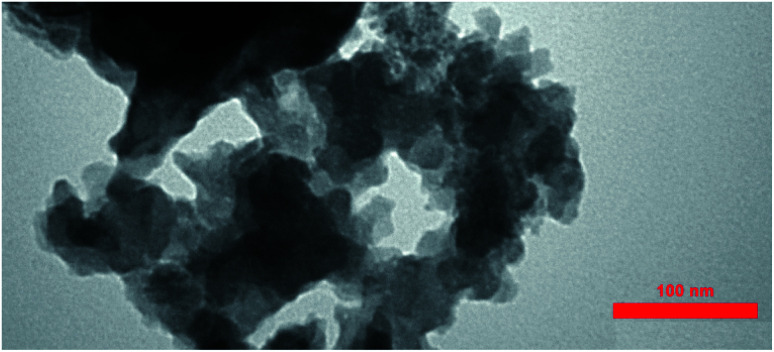
TEM images of the Cu_2_O NPs produced using the *S. latifolium* extract.

The TEM image of the *S. latifolium*-derived Cu_2_O NPs is shown in Fig. 5. As can be seen, the Cu_2_O NPs have irregular shapes and their particle size is lower than 100 nm.

Fig. 4e–f shows the morphology and FESEM images of particles prepared using *P. australis* alga extract. FESEM images clearly present the aragonite spindle-shaped crystals which have sizes of 1–2 μm in length and 300–500 nm in width. On the other hand, it was confirmed that the CaCO_3_ particles practically preserved the spindle/needle-like morphology of the aragonite particles. Therefore, it is concluded that the CaCO_3_ spindle-like particles are successfully fabricated in the presence of *P. australis* algae extract.

#### 2.1.3 FTIR

All brown algae contain a wide range of biologically active such as sulphated polysaccharide, phenolics, fucoxanthin, fucoidan, alginic acid (alginate), laminarin and mannitol.^44–46^ The hydroxyl group (–OH) present in these biostructures makes them a reducing agent in conjunction with high level of secreted enzymes and proteins present in the organic part of the cell extracts.

**Fig. 6:**
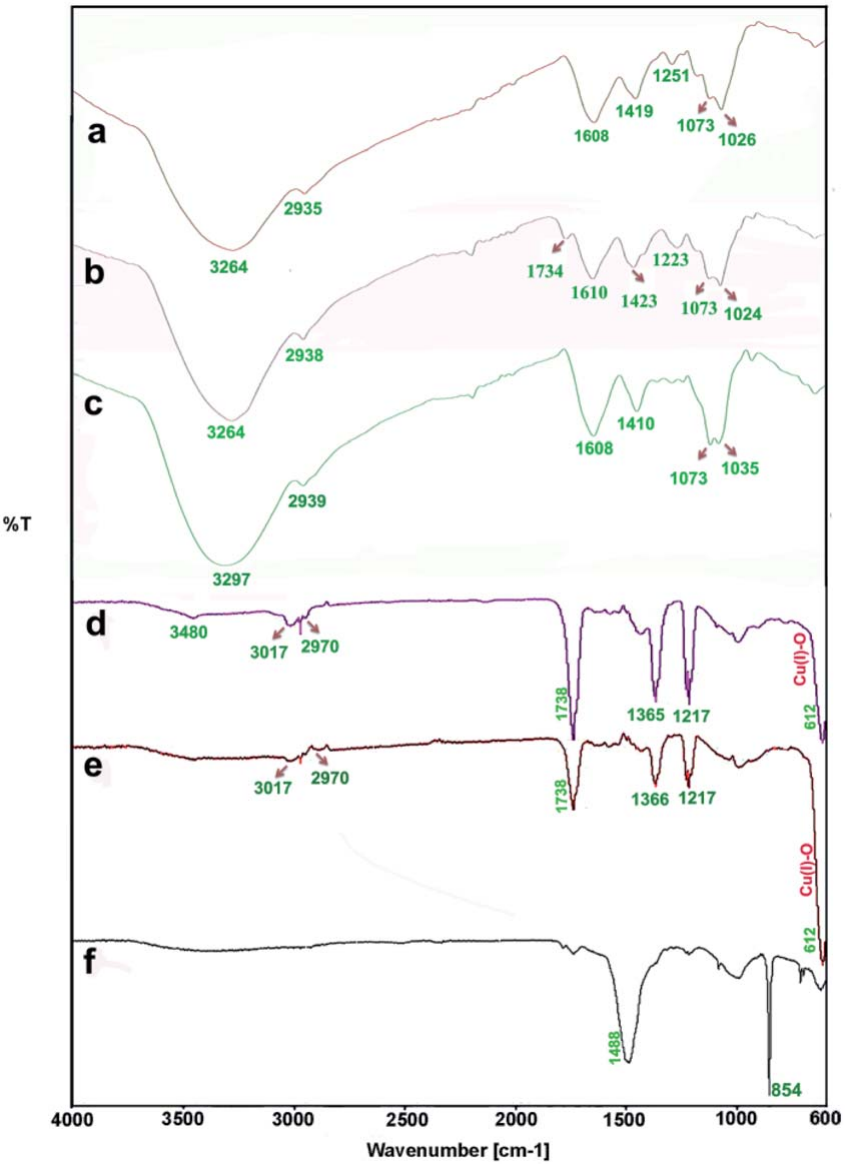
FTIR spectra: the aqueous extracts of *P. australis* (a), *S. latifolium* (b) and *C. myrica* (c); the particles prepared using *C. myrica* (d), *S. latifolium* (e) and *P. australis* (f) extract.

The FTIR measurements were taken to identify the chemical composition and possible biomolecules on the surface of NPs. Fig. 6 shows FTIR spectra of the algae extracts and the as-prepared particles. Generally, the FTIR spectra of the algae extracts consist of notable peaks at around 1734–1608 and 3264–3297 cm^−1^ that may be attributed to the C=O and O–H stretching of compounds, respectively.^47^ The bands at 1410–1423 cm^−1^ and at 2935–2939 cm^−1^ indicate the aliphatic C–H vibrations and the features at 1024–1037 cm^−1^ are representative of the C–O band stretching vibrations.

The both FTIR spectra of samples produced *via C. myrica* and *S. latifolium* are similar, indicating specific peaks of Cu_2_O NPs and capping agents. Characteristic bands of the stretching and bending vibrations of C–H, C=O, C=C and C–O groups attributed to the capping agents and the Cu(i)–O vibration of Cu_2_O are well observed in two spectra. As seen in the FTIR analysis, the absorption bands of the different functional groups associated with the biomaterial supported on the Cu_2_O NPs were shifted to higher wave numbers (cm^−1^) compared to that of the *C. myrica* and *S. latifolium* FTIR spectra. This shift is evidence of the interaction between the Cu_2_O NPs and the stabilizing agent. Also, the FTIR analysis shows a strongly decrease in the peak intensity of the O–H absorption bands of the NPs at 3480, which indicate that the hydroxyl groups of stabilizing agent are the active sites in this system. The bands observed at 3017–2970 cm^−1^ have been assigned to the stretching mode of sp^2^ and aliphatic C–H groups. The strong peak at 1738 cm^−1^ has been assigned to the stretching mode of C=O band. The olefinic C=C bands as the weak absorptions at around 1600 are well seen. The peak at 1366 cm^−1^ is the characteristic band of the aliphatic C–H bending modes. The strong bands observed at 1217 cm^−1^ have been attributed to stretching mode of C–O bands. It was found that all peaks attributed to capping agent obtained by the *C. myrica*-derived NPs have been repeated in the FTIR spectrum of *S. latifolium*-derived NPs with changes in the intensity of absorption bonds. However, the Cu(i)–O vibration of Cu_2_O in both spectra (612 cm^−1^) are relatively similar in the position as well as in the intensity of absorption bonds. Generally, by comparing the spectrum of Cu_2_O NPs produced by *C. myrica* with that of the *S. latifolium*, we can conclude that the two spectra are similar in their spectral features.

The brown algal extracts consist of proteins, carbohydrates, minerals, antioxidants (polyphenols, tocopherols), and pigments such as carotenoids. Fucoxanthin is the main carotenoid produced by brown seaweeds, and it has important benefits for human health including antioxidant, anti-inflammatory and anti-tumour properties.^48–50^ Fucoxanthin through the several olefinic bonds and hydroxyl groups present in its structure can act as a powerful reducing and capping agent in the algae-mediated construction of NPs. Negm *et al.*^47^ have reported the xanthophyll pigment (fucoxanthin) as the main component of the brown algae to interact with metal ions. They confirmed that fucoxanthin has the ability to interact with the metal ions throughout the electron donating groups and plays an important role in adsorption of the metal ions from the medium, which can explain the higher adsorption efficiencies of the brown algae than the mushroom as biosorbent.

According to the FTIR measurements, we believe that the bio-compound supported on the surface of Cu_2_O NPs has a very close chemical composition to the fucoxanthin,^51^ and in this work, fucoxanthin is the main component in the *C. myrica* and *S. latifolium* extract to stabilize the Cu_2_O NPs. Meanwhile, definite and exact mechanism of the reduction stage and Cu_2_O NPs formation has not been thoroughly explored due to the diversity of compounds available in a marine brown alga.

As shown in Fig. 6f, the FTIR spectrum of the sample produced using *P. australis* extract has depicted characteristic carbonate infrared vibrations for aragonite structures.^40,52^ This spectrum displays characteristic absorption bands out-of-plane bending (*υ*_2_) at 854 cm^−1^ and the vibrations of doubly degenerate planar bending (*υ*_4_) at 713 cm^−1^ along with a weak 700 cm^−1^ absorption peak. A characteristic doubly degenerate planar asymmetric stretching vibration (*υ*_3_) is also seen at 1488 cm^−1^. Therefore, the FTIR analysis confirms the formation of aragonite CaCO_3_ using *P. australis* extract and these FTIR observations are considered as the common characteristic features of the CO_3_^2−^ ions in CaCO_3_ and are the fundamental modes of vibration for this molecule. The characteristic bands of the O–H, C–H and C–O attributed to *P. australis* extract as weak absorption were also observed in the FTIR spectrum.

#### 2.1.4UV-vis

**Fig. 7:**
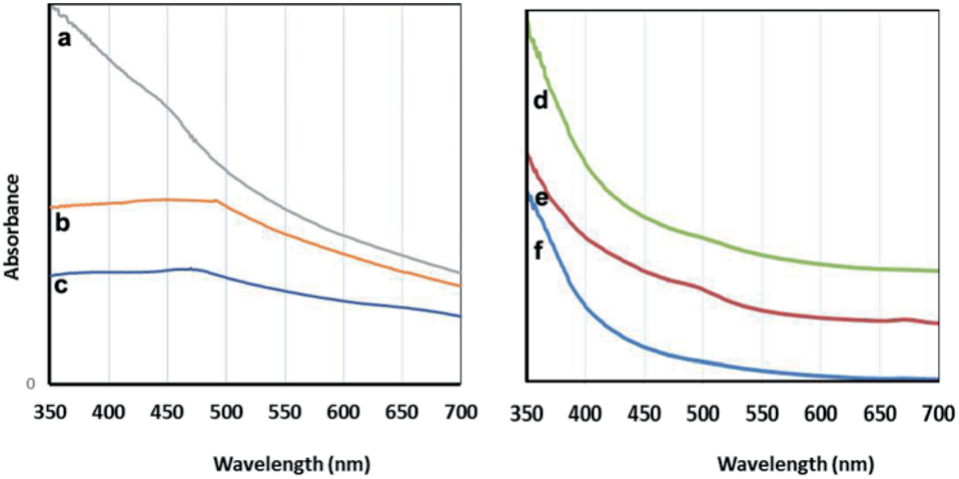
Visible spectra: the particles prepared using *C. myrica* (a), *S. latifolium* (b) and *P. australis* (c); the aqueous extracts of *C. myrica* (d), *P. australis* (e) and *S. latifolium* (f).

To investigate the optical properties, visible spectra of the aqueous algae-extracts and the algae-mediated particles were examined and are depicted in Fig. 7. As seen in the absorbance spectra, the maximum absorption wavelengths of the *C. myrica* and *S. latifolium*-derived Cu_2_O NPs were observed at 475 nm and 491 nm, respectively, whereas the Vis-spectra of the *C. myrica* and *S. latifolium* extracts do not show any absorption band in this region.

It is well-known that the absorption bands of nanoparticles are affected by the quantum size effect (QSE), morphology, and crystallinity.

It is well known that adding different amounts of sodium citrate and sodium carbonate to a blue solution of CuSO_4_ causes deep blue copper-citrate complexes to produce.^53^ Some reports have confirmed that polysaccharides and polymers having hydroxyl groups in their chains form complexes with Cu(ii) ions that this polymer–copper complexes are green in color.^14,54^ Accordingly, we believe that the appearance of green color after adding the algae extract into the deep blue copper(ii)-citrate solution may be due to complex formation of copper(ii) ions with hydroxyl groups of polysaccharides and other OH-functionalized contents of aqueous algae extract. In two cases *C. myrica* and *S. latifolium*, this color change and green complex formation occurs more strongly than that for *P. australis*. Therefore, it can be stated that the reduction stage takes place in green complex network, and the copper(ii) ions which are chelating with OH groups of algae extract are reduced and Cu_2_O NPs are successfully formed. All these observations are related to the contents of the algae extract. Based on previous reports,^55^ it is worth noting that the crude extract of algae provides both the capping and reducing agent(s) and can also act as a size, composition and shape controlling factor for synthesis of nanoparticles. Indeed, biomolecules contained within either algae extract act as natural stabilizing agents on specific facets of the forming crystal.

In this study for three samples, the biosynthesis involves three steps: preparation of (i) algal extract, (ii) metal precursor solution, and (iii) exposure of algal extract to metal precursor solution. In the first step of the reaction process, the liquid algae extract is mixed with the solution of metal precursor. In the case of *C. myrica* and *S. latifolium*-mediated synthesis the colour change of the reaction mixture is an evidence of the reaction initiation. After nucleation and particle growth process, the thermodynamically stable nano-sized Cu_2_O particles are formed in reaction media. The bioactive components of extract are effective as the reducing and stabilizing agent and promote the Cu_2_O NPs formation.

### 2.2. Antibacterial tests

**Fig. 8:**
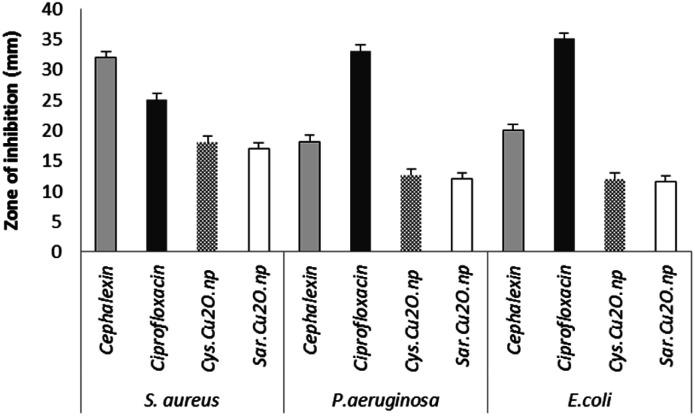
The d of antibacterial disk diffusion test of Cu_2_O NPs synthesized using *S. latifolium* (Sar) and *C. myrica* (Cys) based on the diameters of zone of inhibition (mm).

The antibacterial activity of as-synthesized Cu_2_O NPs was examined against both Gram-negative and Gram-positive bacteria by using disc diffusion test. The results of disk diffusion susceptibility tests are summarized in Fig. 8. The presence of the zone of inhibition more than 10 mm around the Cu_2_O NPs disks confirmed the antibacterial activity of these nanoparticles synthesized by *S. latifolium* and *C. myrica* (*p* < 0.05). The highest zone of inhibition diameter was resulted against *S. aureus* by Cu_2_O NPs synthesized by *C. myrica* (18 ± 1.51 mm) and synthesized by *S. latifolium* (17 ± 1.49 mm), while the zone of inhibition against *E. coli* caused by Cu_2_O nanoparticles synthesized by *S. latifolium* was 11.5 ± 1.13 mm. However, no significant difference was observed in the antibacterial activity between *S. latifolium* and *C. myrica* synthesized Cu_2_O NPs. This clearly demonstrates that these algae-based Cu_2_O NPs have respectable antimicrobial activity against both *E. coli* (Gram-negative) and *S. aureus* (Gram-positive). However the results also show that the antibacterial activity against *S. aureus* (*p* < 0.05) is significantly higher than that against *E. coli*, probably due to cell walls structure difference between Gram-positive and Gram negative bacteria. The cell wall of the Gram-negative consists of proteins, lipids and lipopolysaccharides (LPS) that provide effective protection against antibacterial material whereas that of the Gram-positive does not consists of LPS.^56^

The disk diffusion test results are considered as primary screening evidence of antibacterial activity; hence the complementary tests of MIC/MBC were performed. The susceptibility of *Staphylococcus aureus* to as-synthesized Cu_2_O NPs was evidences by MIC/MBC of (125/250 μg mL^−1^) for Cu_2_O NPs produced by both *S. latifolium* and *C. myrica* algae (Table 1). No significant difference was observed in antibacterial effect of Cu_2_O nanoparticles synthesized using *S. latifolium* and *C. myrica* (*P* < 0.05). These values are in agreement with some of the previous experiments on antibacterial properties of copper oxide nanoparticles. Ghadiri *et al.* reported the zone of inhibition between 17–20 mm for Cu_2_O NPs, however no considerable bacterial inhibition was demonstrated for CuO NPs.^57^ However, different zone of inhibitions and MIC/MBC values were attributed to the size, shape and distribution of nanoparticles in addition to the pH of culture media. Bezza *et al.* reported dose and pH-dependent bactericidal activity of Cu_2_O NPs.^37^ They demonstrated higher antibacterial effect of particles with narrow size distribution compared to larger nanoparticles. The smaller size of nanoparticles and the higher surface area to volume ratio influence the antibacterial potential of copper compounds due to more effective contact and reactive oxygen species release.

**Table 1:** Minimum inhibitory concentration and minimum bactericidal concentration of Cu_2_O synthesized using *S. latifolium* and *C. myrica* against *Staphylococcus aureus*, *Escherichia coli* and *Pseudomonas aeruginosa*

Cu_2_O NPs	Bacteria	MIC (μg mL^−1^)	MBC (μg mL^−1^)
Cu_2_O synthesized using *S. latifolium*	*Staphylococcus aureus*	125	250
	*Escherichia coli*	500	500
	*Pseudomonas aeruginosa*	500	500
Cu_2_O synthesized using *C. myrica*	*Staphylococcus aureus*	125	250
	*Escherichia coli*	250	250
	*Pseudomonas aeruginosa*	250	250

Nanostructured materials have received a considerable attention for their antibacterial and biological properties in the recent years.^28–31,58^ Due to their high potential and advantages such as long shelf life over common antibiotics and cytotoxic compounds many studies conducted on nanostructured copper-based particles.^59–61^ The antibacterial activity of other forms of copper-based nanoparticles was verified in previous studies, and researches confirm the effect of nanoparticle size on antibacterial activity.^62^ Therefore the copper derived nanoparticles show a high promise to new metal based antibiotics.

A number of research groups have investigated the antibacterial mechanism of metal oxide nanoparticles. Based on some reports, the antibacterial activity of metal oxides is generally attributed to immediate disruption in integrity of cell membrane and generation of reactive oxygen species (ROS).^30,37,63^ Copper can participate in several chemical reactions, which lead to the formation of the highly reactive oxygen species (ROS) and hydroxyl radical intermediates.^64^ Ikram *et al.* have recently investigated the antibacterial activity of a variety of copper-assisted nanoparticles.^28,35,65^ They have stated that Cu-doped ZnO nanorods as antibacterial agent produce reactive oxygen species (ROS) on the bacterial cell membrane, which resulted in the extrusion of cytoplasmic contents and the bacteria death. They have also suggested another possible mechanism for the action of nanomaterials antibacterial in which the cations (Cu^2+^ and Zn^2+^) form strong interactions with the negatively charged parts of the bacterial cell membrane, which leads to a collapse of the micro pathogens. Bezza *et al.* have studied the antibacterial mechanism of Cu_2_O NPs and examined the effect of the NPs on the bacterial cell morphology and changes in the cellular ultrastructure.^37^ They found that the Cu_2_O NPs attach to the bacterial body with associated cell wall decomposition and cytoplasmic membrane rupture which leads to the outflow of internal cellular contents, collapse and death of cells. However, recent works suggest additional toxicity mechanisms by which cell damage and resulting cell death occur, mainly through replacement of iron by copper in iron-sulphur (Fe–S) cluster proteins.^37,66,67^ Induction of cellular oxidative stress can result in irreversible cell damages by disruption in a series of functional and structural mechanisms such as inhibition of cell respiration, lipid peroxidation and membrane instability in addition to oxidative damage to proteins and nucleic acids.

However, the comparative researches and detailed observation on the mechanism of bactericidal and cytotoxic action in addition to precise study on environmental and side effects of metal nanoparticles are recommended before commercialization.

### 2.3. Cytotoxicity assessment of biologically synthesized Cu_2_O NPs

**Fig. 9:**
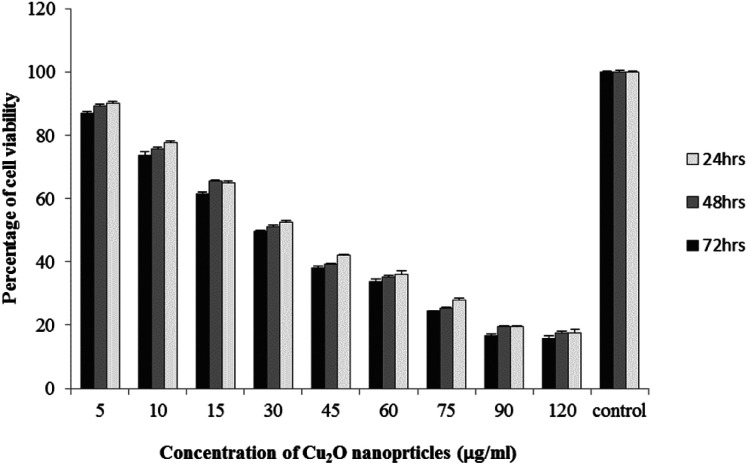
MTT assay results of human leukemia cell line (K562) treated to Cu_2_O NPs biosynthesized by *C. myrica* extract.

**Fig. 10:**
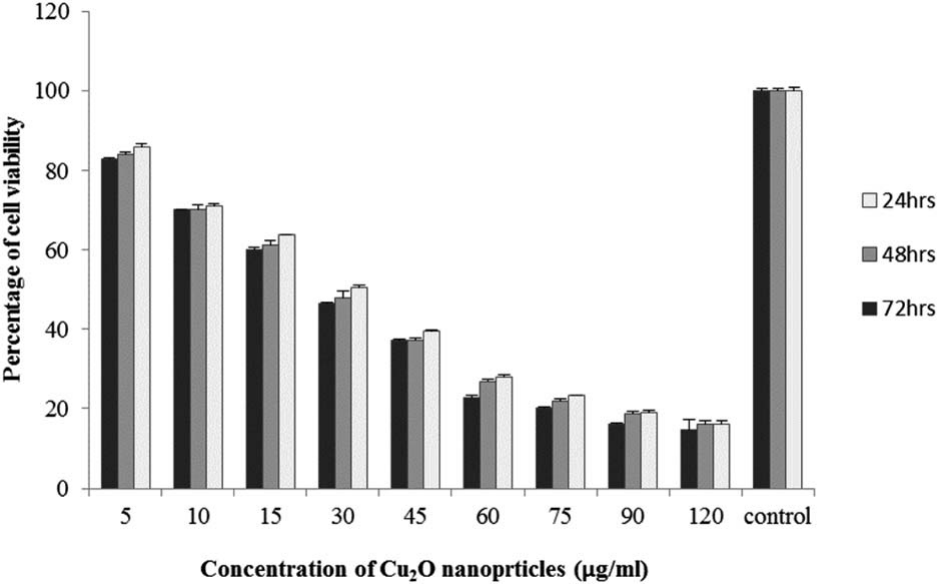
MTT assay results of human leukemia cell line (K562) treated to Cu_2_O NPs biosynthesized by *S. latifolium* extract.

**Fig. 11:**
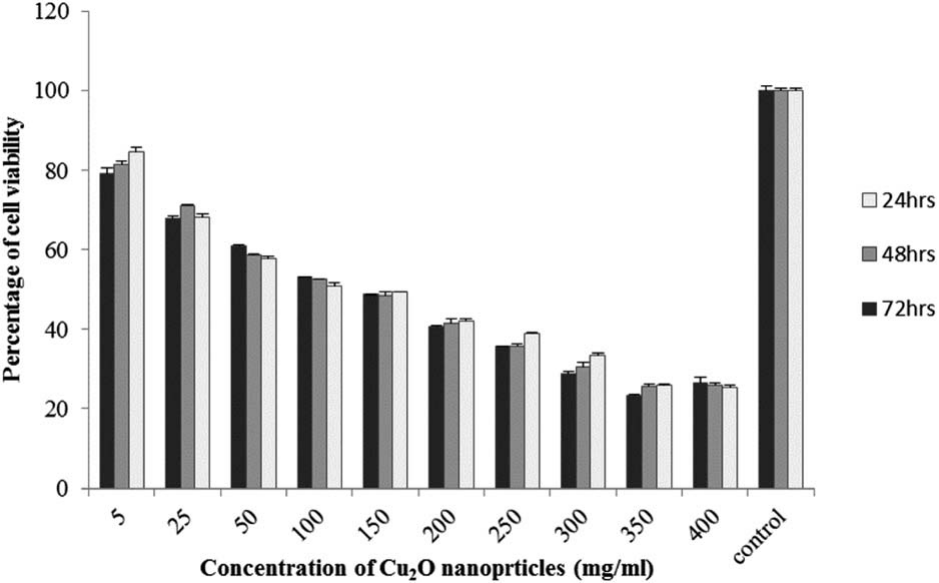
MTT assay results of human dermal fibroblast (HDF) treated to Cu_2_O NPs biosynthesized by *C. myrica* extract.

**Fig. 12:**
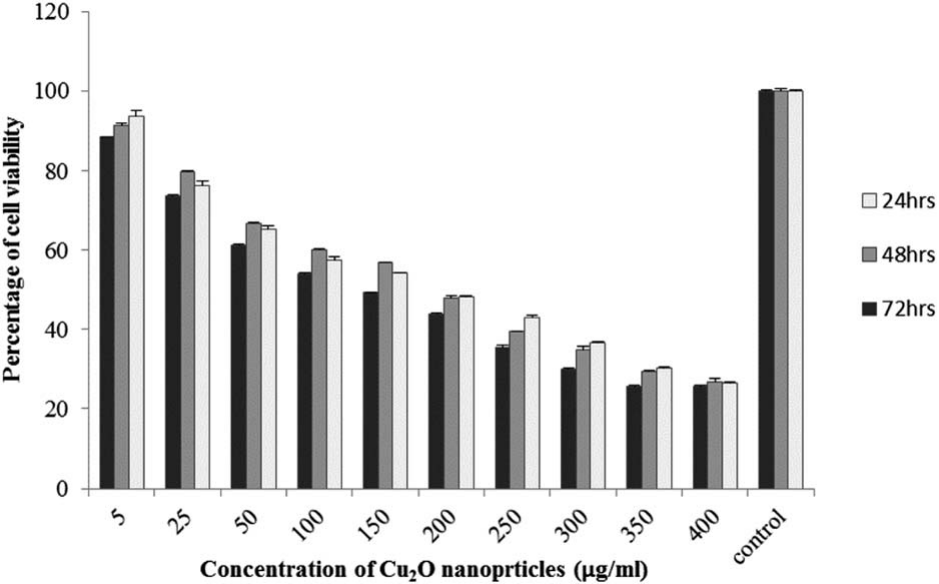
MTT assay results of human dermal fibroblast (HDF) treated to Cu_2_O NPs biosynthesized by *S. latifolium* extract.

The results of viability test obtained from exposing the K562 and HDF to different concentrations of biosynthesized Cu_2_O NPs nanoparticles in 24, 48 and 72 hours are summarized in Fig. 9–12. All treated concentrations of biosynthesized Cu_2_O NPs (using *S. latifolium* and *C. myrica* extracts) showed significant decrease in cell viability (*P* < 0.05). However, the growth inhibition effect of nanoparticles was lower on HDF normal cells (*p* < 0.05). The cytotoxicity of cells increased in correlation to concentration of biosynthesized nanoparticles (*P* < 0.05). No significant differences was observed between the times of incubation (24, 48 and 72 hours). The toxicity effect of biosynthesized Cu_2_O NPs on viability of human leukemia cell line (K562) was significant (*P* < 0.05). The effect of nanoparticles on human dermal fibroblast normal cells was lower in comparison to human leukemia cell line (*P* < 0.01), as the viability of HDF even at higher concentrations of 400 μg mL^−1^ remained higher than 20% whereas the viability of K562 decreased under 20% at concentration of 120 μg mL^−1^. The IC_50_ of CuO_2_(i) nanoparticles was calculated at 30 mg mL^−1^ and no significant difference was observed in IC_50_ of Cu_2_O NPs extracted by *C. myrica* and *S. latifolium* on K562 cells; however, the IC50 of Cu_2_O on normal cells of HDF was found different for nanoparticles synthesized by *S. latifolium* (200 μg mL^−1^) and *C. myrica* (150 μg mL^−1^). No significant difference was observed between 24, 48 and 72 hours of exposure to Cu_2_O NPs at IC50 concentration of 30 μg mL^−1^.

Despite the widespread studies focusing on the cytotoxicity of Ag and Au nanoparticles, a few studies are available on less expensive compounds such as copper oxide, especially the biosynthesized forms. The results of the present study confirmed the significant K562 cancer cell growth inhibition at low tested concentrations (above 5 μg mL^−1^). The Cu_2_O NPs biosynthesized using *S. latifolium* and *C. myrica* showed a higher toxicity effect on human leukemia cell line (K562) (IC50 = 30 μg mL^−1^) compared to normal human dermal fibroblast (*p* < 0.05). Our results are in agreement with recent studies which have approved cytotoxicity of metal nanoparticles.^61,68–70^ Present study exhibits high potentiality of Cu_2_O for its anticancer activity as confirmed by concentration dependent cytotoxicity of Cu_2_O nanoparticles on K562 cells in addition to lower toxicity to normal cells. However, several researches are underway to investigate the mechanism of Cu_2_O action and possible pathways of cytotoxicity and their side effects.

## 3. Conclusions

Metal oxide nanoparticles are commonly synthesized by physical and chemical methods, where the different chemicals are utilized as reducing and stabilizing agents. As an alternative, biological synthesis of metal oxide nanoparticles using natural living organisms such as plants, algae, and microbes has recently emerged as a green, cost-effective and safe method. Cuprous oxide (Cu_2_O) is a metal oxide semiconductor attracting great attention in various fields of science and technology, including medicine, solar energy, catalysis and environmental practices. Here, we successfully synthesized pure and stable Cu_2_O nanoparticles by a simple, mild and eco-friendly method using water extract of *C. myrica* and *S. latifolium* algae as the reducing and stabilizing agent. Many of the biomolecules present in the cell walls of these brown algae can act as capping agents and biocatalysts to assist in the reduction of Cu^2+^ ions to Cu^1+^. However, this reaction in the presence of *P. australis* extract didn't produce any Cu_2_O nanoparticles, and spindle shaped CaCO_3_ were prepared. The algae-derived Cu_2_O nanoparticles were well characterized and examined as antibacterial and anticancer agents. The antibacterial activity of nanoparticles against Gram-positive bacteria (*Staphylococcus aureus*) was obtained to be significantly higher than that against Gram-negative bacteria (*Pseudomonas aeruginosa* and *Escherichiacoli*). Furthermore, the Cu_2_O nanoparticles showed an effective anticancer activity tested on human leukemia cell line (K562).

Algae are reservoirs of important natural products and will attract the attention of many researchers in the near future. A considerable boom may be observed in the algae-mediated biosynthesis of NPs, which will be likely to have great potentials in many aspects of science and technology.

## 4. Experimental

### 4.1 General remarks

All reagents were purchased from commercial suppliers (Merck and Aldrich) and used without further purification. The FT-IR spectra of products were measured in ATR approach by JASCO FTIR-4100 spectrophotometer. The X-ray diffraction (XRD) patterns were recorded by using a Philips Xpert MPD diffractometer with Cu Ka radiation (*l* = 0.15418 nm). Transmission Electron Microscopy (TEM) was carried out on Philips EM 208S 100KV. Scanning electron microscopy (FE-SEM) images were obtained using a Mira 3-XMU instrument. The UV-vis spectra were recorded on a Shimadzu UV-2100 spectrometer.

### 4.2. Preparation of alga extract

The algae were harvested from the Bushehr coast of Persian Gulf (southwestern Iran) and cleaned carefully in fresh water and then by distilled water to remove sand and salts. The cleaned brown algae were dried at ambient temperature and crushed into powder. About 5 g of powdered algae was separately transferred into a 250 mL beaker containing 200 mL double distilled water, and the mixture was boiled with a magnetic stirrer for 20 min. The solutions were then allowed to cool at room temperature and the extracts obtained was filtered and used as a reaction agent and stabilizer.

### 4.3. Synthesis of copper(i) oxide nanoparticles using *S. latifolium* and *C. myrica* extract

At first, a primary solution was prepared by dissolving 1 g CuSO_4_·5H_2_O, 5 g sodium citrate and 2.5 g of sodium carbonate in 200 mL double distilled water. Then, 6 mL of freshly prepared algal extract was added dropwise into 100 mL of above primary solution under vigorous stirring at 100 °C. After several seconds, the deep blue solution gradually changed to green and within few minutes (15 min and 20 min for solutions containing *S. latifolium* and *C. myrica* extract, respectively) the orange-red Cu_2_O nanoparticles appeared in the greenish reaction mixture. The resulting reddish products were isolated by centrifugation, washed thoroughly with distilled water several times to remove impurities and dried in air.

The reaction in the presence of *P. australis* extract was done in the same way as for the *S. latifolium* and *C. myrica* extract, but no Cu_2_O nanoparticles were formed.

### 4.4. Antibacterial activity of biosynthesized Cu_2_O NPs

#### 4.4.1. Preparation of bacteria

The antibacterial activity of green synthesized nanoparticles was tested against three pathogenic bacteria *Staphylococcus aureus* PTCC 1112 (Gram positive), *Escherichia coli* PTCC1330 (Gram negative) and *Pseudomonas aeruginosa* PTCC1310 (Gram negative). Bacteria were obtained from Iranian Biological Resource Center, Tehran, Iran, pre-cultured in Muller Hinton broth (Merck, Germany) at 37 °C for 24 h and agitated at 200 rpm.

#### 4.4.2. Disk diffusion susceptibility test

The susceptibility of bacteria to Cu_2_O nanoparticles was tested using Kirby–Bauer disk diffusion method.^71^ A 0.5 McFarland standard (1/5 × 10 CFU mL^−8^) of bacterial turbidity was prepared, and each bacterial strain was uniformly swabbed on Muller-Hinton agar medium. Blank disks were loaded with 30 μL (600 μg mL^−1^) of Cu_2_O NPs, and cephalexin (20 μg mL^−1^) and ciprofloxacin (20 μg mL^−1^) antibiotics (as positive control). After 24 hours of incubation of disks on agar plate at 37 °C the zone of inhibition was measured.

#### 4.4.3. Minimum inhibitory concentration (MIC) and minimum bactericidal concentration (MBC)

The MIC of Cu_2_O nanoparticles synthesized by two algae *S. latifolium* and *C. myrica*, was measured by culture of bacteria in 96 well microplate using standard microdilution in Muller-Hinton broth based on Clinical & Laboratory Standards Institute (2012). Minimum bactericidal concentration (MBC) was taken as the lowest bactericidal concentration of Cu_2_O NPs in which 99.9% of bacteria could not survive.^72^ For this, bacteria were taken from MIC test in suspension and cultured on Muller Hinton agar plates at 37 °C for 24 h.

### 4.5. Anticancer activity of Cu_2_O NPs

#### 4.5.1. Cell viability tests

Human dermal fibroblasts (HDF) and K562 human leukemia cell line were obtained from Iranian Biological Resource Center, Tehran, Iran. Cells were maintained in RPMI 1640 (Merck, Germany) or (Hyclone SH30027.LS). Culture media were supplemented with 10% Fetal Bovine Serum (Gibco, Life Technologies, Inc., New York, USA), and 5% of penicillin/streptomycin (Sigma Aldrich St. Louis, MO, USA). Cells were culture in a 5% CO_2_ humidified atmosphere at 37 °C.

Cell proliferation was assessed using MTT (3-[4,5-dimethylthiazol-2-yl]-2,5-diphenyl tetrazolium bromide) assay. This assay is based on measuring the mitochondrial activity of cells which assessed by the conversion of the tetrazolium salt into formazan crystals. Mitochondrial activity is linearly related to number of viable cells. For this, cells were incubated for 24 hours in 96 well cell culture plates at an approximate concentration of 5 × 10^3^ cells. Both HDF and K562 cells then were treated to series of *S. latifolium* and *C. myrica* biosynthesized Cu_2_O nanoparticles along with cell control. Threated cells were incubated for 72 hours and MTT assay was carried out in 24, 48 and 72 hours. MTT solution (5 mg mL^−1^ prepared in PBS) was added to each well and after 4 hours of incubation at 37 °C, 100 μL of dimethyl sulphoxide (DMSO) was added and absorbance was measured at 490 and 570 nm by spectrophotometry using plate reader (Eliza MAT 2000, DRG Instruments, GmbH). Percentages of viability were calculated as the percentage of treated cell viability (OD) in relation to experimental control.

#### 4.5.2. Statistical analysis

The statistical analysis was performed using SPSS software Version 16 (SPSS Inc., Chicago, IL, USA). The significance of differences among experimental groups and control group was calculated using one-way ANOVA, and data expressed as mean ± S. E. and accepted at a statistical significance level of *P* < 0.05.

## Conflicts of interest

All authors declare that they have no conflict of interest associated with this publication.

## Supplementary Material
